# Enhanced Performance of PECVD-Grown β-Ga_2_O_3_ MSM Solar-Blind Ultraviolet Photodetectors via a SiO_2_ Buffer Layer

**DOI:** 10.3390/nano16211403

**Published:** 2026-11-01

**Authors:** Yajun Tian, Lingsen Meng, Qijin Cheng

**Affiliations:** School of Electronic Science and Engineering, Xiamen University, Xiamen 361102, China; yajun__tian@163.com (Y.T.); lingsenmeng@stu.xmu.edu.cn (L.M.)

**Keywords:** β-Ga_2_O_3_, PECVD, heteroepitaxy, SiO_2_ buffer layer, solar-blind ultraviolet photodetector

## Abstract

Heteroepitaxial growth of β-Ga_2_O_3_ thin films on Si for solar-blind ultraviolet photodetectors faces severe challenges due to lattice mismatch and interfacial oxidation, leading to high dark current and low responsivity. In this work, β-Ga_2_O_3_ thin films were deposited on n-Si(100), p-Si(100), and p-Si(100) substrates with a 100 nm thermally oxidized SiO_2_ buffer layer using plasma-enhanced chemical vapor deposition. Metal-semiconductor-metal structured solar-blind ultraviolet photodetectors were subsequently fabricated. The effects of the SiO_2_ buffer layer on the crystalline quality, interfacial characteristics, and optoelectronic performance of the devices were systematically investigated. Results show that the SiO_2_ buffer layer possibly relieves the mismatch-induced stress in Ga_2_O_3_ films on Si, suppresses the formation of an uncontrollable interfacial layer tentatively assigned as SiO*_x_*-like (*x* ≤ 2) during the initial deposition stage, and significantly improves the crystalline quality of β-Ga_2_O_3_ films. Compared with devices on bare Si substrates, the photodetector based on the SiO_2_-buffered substrate exhibits a significantly reduced dark current, a photo-to-dark current ratio of 5.01 × 10^3^, a responsivity of 3.32 A/W, a theoretical shot-noise-limited specific detectivity of 1.14 × 10^13^ Jones, and a UV-to-visible rejection ratio as high as 8.12 × 10^5^ at 5 V. The overall performance of the SiO_2_-buffered device is markedly superior to that of bare Si devices, albeit with a slight decrease in response speed. Mechanism analysis reveals that the dense SiO_2_ buffer layer blocks the vertical leakage path of carriers toward the Si substrate, simultaneously relieving the mismatch-induced stress and improving film quality. This work provides a feasible technical pathway toward low-cost, CMOS-compatible high-performance silicon-based solar-blind ultraviolet photodetectors.

## 1. Introduction

Silicon (Si), as the cornerstone of the microelectronics industry, offers unparalleled advantages including low cost, large wafer size, high thermal conductivity, and mature processing technologies. Therefore, heterogeneous integration of Ga_2_O_3_ thin films with Si substrates not only significantly reduces device cost but also enables monolithic integration of high-performance solar-blind ultraviolet detection functions with advanced silicon-based complementary metal-oxide-semiconductor (CMOS) readout circuits. This holds great appeal for complex system-on-chip applications such as intelligent sensing and ultraviolet imaging [1,2,3]. However, the direct growth of high-quality β-Ga_2_O_3_ thin films on Si substrates faces substantial challenges. First, the lattice mismatch exceeding 20% between Ga_2_O_3_ and Si, along with significant differences in thermal expansion coefficients, results in high densities of dislocations and defects in the epitaxial films, and even film cracking [4]. Second, in an oxygen-rich growth environment, interfacial reactions between Ga_2_O_3_ and the Si substrate readily occur during the initial deposition stage, generating an uncontrollable amorphous SiO*_x_* or silicate layer [5,6,7,8]. This interfacial layer introduces a large number of defect states, severely affecting carrier transport and degrading device performance.

Recent reviews have summarized the rapid progress of Ga_2_O_3_ solar-blind photodetectors, covering material growth, device architectures, and applications ranging from civilian sensing to missile detection [9,10]. To date, however, research on the heteroepitaxial growth of Ga_2_O_3_ on Si substrates remains relatively limited, with most studies still focusing on lattice-matched substrates such as sapphire and MgO. In existing studies, researchers have primarily employed materials such as AlN [11,12,13], Al_2_O_3_ [14,15], HfO_2_ [16], or utilized seed layers [17,18] and post-annealing processes [4] to mitigate the lattice and thermal mismatches between Ga_2_O_3_ and Si. For example, Gao et al. [12] significantly improved the crystalline quality of β-Ga_2_O_3_ thin films and reduced the photodetector’s dark current by introducing an AlN buffer layer; Liu et al. [16] inserted an Al_2_O_3_ or HfO_2_ buffer layer using atomic layer deposition, effectively suppressing thermally induced flower-like cracking defects on the surface of Ga_2_O_3_ thin films grown on Si substrates and reducing surface roughness; Arora et al. [17] achieved a transition of Ga_2_O_3_ thin films from amorphous to polycrystalline by introducing a high-temperature seed layer, and fabricated high-performance self-powered solar-blind ultraviolet photodetectors with a higher photo-to-dark current ratio and lower dark current. Although these methods have achieved certain progress, they typically require additional deposition equipment and complex process steps, increasing fabrication cost and process complexity. In contrast, selecting SiO_2_ as a buffer layer offers unique advantages. First, SiO_2_ can be directly generated by the in situ thermal oxidation of Si substrates, featuring simple processing and low cost. Second, thermally oxidized SiO_2_/Si substrates are standard substrates in CMOS technology; their use facilitates monolithic integration of Ga_2_O_3_-based photodetectors with silicon-based signal processing circuits. Third, the dense SiO_2_ layer can act as an effective diffusion barrier, physically isolating Ga_2_O_3_ from the Si substrate and suppressing interfacial reactions and elemental interdiffusion during growth. This leads to the grown Ga_2_O_3_ thin films having fewer interfacial defects and higher crystalline quality.

Based on the aforementioned analysis, in this work, β-Ga_2_O_3_ thin films are deposited on three types of substrates—namely, n-Si(100), p-Si(100), and p-Si(100) with a 100 nm thermally oxidized SiO_2_ layer on one side—using plasma-enhanced chemical vapor deposition (PECVD). Metal-semiconductor-metal (MSM) structured solar-blind ultraviolet photodetectors are subsequently fabricated. The effects of substrate doping type and the introduction of a thermally oxidized SiO_2_ buffer layer on the crystalline structure, surface morphology, optical properties, and optoelectronic performance of the Ga_2_O_3_ thin films and corresponding devices are systematically investigated. By incorporating the thermally oxidized SiO_2_ buffer layer, we aim to achieve high-quality β-Ga_2_O_3_ thin films on silicon substrates, optimize the dark current and photoresponse characteristics of the devices, and provide a feasible technological pathway toward low-cost, high-performance, CMOS-compatible silicon-based solar-blind ultraviolet photodetectors.

## 2. Experimental Section

### 2.1. Growth of Ga_2_O_3_ Thin Films

β-Ga_2_O_3_ thin films were deposited using a PECVD system (model BTF-1200C-PECVD-500A, BEQ Equipment Technology Co., Ltd., Hefei, China). High-purity metallic gallium (Aladdin Biochemical Technology Co., Ltd., Shanghai, China; 99.99%) served as the gallium source, with high-purity oxygen (99.999%) as the oxidizing gas and high-purity argon (99.999%) as the carrier/protective gas. In this work, three different types of silicon-based substrates were selected: n-Si(100), p-Si(100), and p-Si(100) with a single-sided 100 nm SiO_2_ thermal oxide layer. Prior to film deposition, the p-Si(100) substrate with a single-sided 100 nm SiO_2_ thermal oxide layer only underwent standard cleaning, which involved ultrasonic cleaning in acetone, ethanol, and deionized water for 15 min each, followed by drying with a nitrogen gun. For the n-Si(100) and p-Si(100) substrates, in addition to standard cleaning, RCA cleaning and removal of the native oxide layer were also performed.

**Figure 1:**
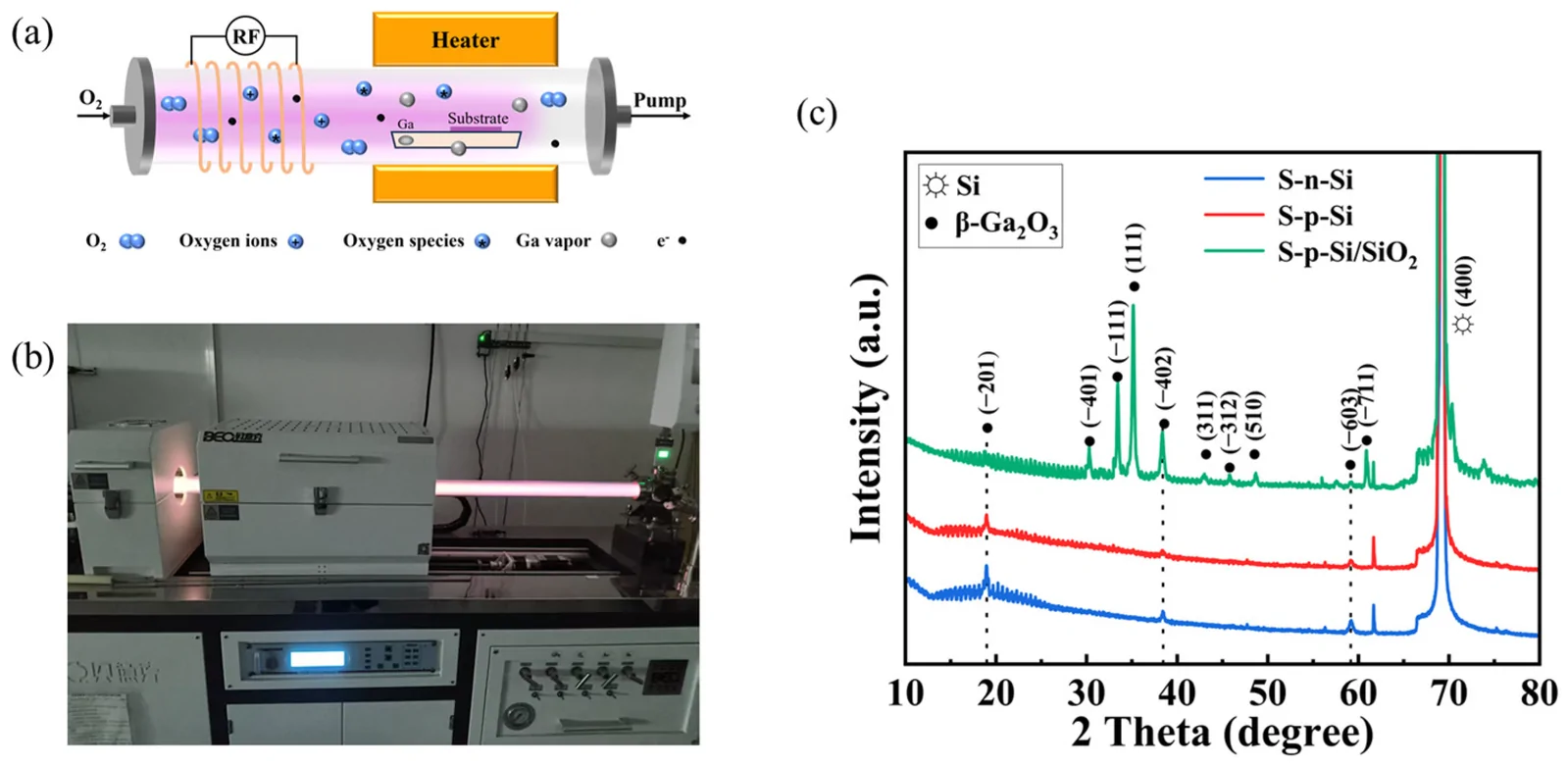
(**a**) Schematic diagram of the gallium oxide deposition via PECVD. (**b**) A typical glow discharge of PECVD system under an RF plasma power of 130 W and an oxygen flow rate of 30 sccm. (**c**) XRD patterns of Ga_2_O_3_ thin films prepared on different types of substrates by PECVD.

Figure 1a shows a schematic diagram of the gallium oxide deposition via PECVD. A 0.3 g gallium metal pellet was placed on one side of a porcelain boat, with the silicon-based substrate positioned on the opposite side (downstream of the precursor gas flow). Growth was conducted at 850 °C with a radio-frequency (RF) plasma power of 130 W. During the heating and cooling stages, the argon flow rate was maintained at 50 sccm. Upon reaching the target temperature, the argon flow was stopped, and oxygen was introduced at a flow rate of 30 sccm for 90 min. Figure 1b shows a typical glow discharge of the PECVD system under an RF plasma power of 130 W and an oxygen flow rate of 30 sccm.

It is noteworthy that during growth, Ga vapor generated at 850 °C was transported by the carrier gas and adsorbed onto the substrate surface. RF-excited active oxygen species arrived at and migrated across the substrate surface, enabling adsorbed Ga atoms to react with active oxygen species to form nuclei. Continuously adsorbed species promoted the expansion of nuclei into two-dimensional Ga_2_O_3_ islands, ultimately forming continuous films. The resulting Ga_2_O_3_ thin films were labeled as S-n-Si, S-p-Si and S-p-Si/SiO_2_, corresponding to n-Si(100), p-Si(100), and p-Si(100) with a single-sided 100 nm SiO_2_ thermal oxide layer, respectively.

### 2.2. Device Fabrication

Ga_2_O_3_ thin films were fabricated into photodetectors with a finger-type metal-semiconductor-metal (MSM) structure using photolithography and electron beam evaporation techniques. The length, width, and spacing of the fingers were 100, 10, and 10 μm, respectively. The electrodes consisted of Ni and Au bimetal layers, with a thickness of 100 and 50 nm, respectively. Specifically, Ni is used to form Schottky contact with Ga_2_O_3_, and Au serves as the electrode protective layer. The fabricated devices are designated as PD-n-Si, PD-p-Si, and PD-p-Si/SiO_2_, corresponding to the three different substrates of n-Si(100), p-Si(100), and p-Si(100) with a single-sided 100 nm SiO_2_ thermal oxide layer, respectively.

### 2.3. Characterization Equipment

The crystal structure and phase composition of Ga_2_O_3_ thin films were measured by X-ray diffraction (XRD, Shimadzu XRD-7000, Shimadzu, Tokyo, Japan) with Cu *K*_α_ radiation (λ = 1.5405 Å). The surface roughness of Ga_2_O_3_ thin films was studied using an atomic force microscope (AFM, Asylum Research Cypher S, Oxford Instruments Asylum Research, Santa Barbara, CA, USA) with a scanning area of 5 μm × 5 μm conducted in the tapping mode. The surface morphology and cross-sectional images of β-Ga_2_O_3_ thin films were obtained using a field emission scanning electron microscope (FESEM, Zeiss Supra 55, Carl Zeiss, Oberkochen, Germany). The fine spectra of O 1*s* were measured using X-ray photoelectron spectroscopy (XPS, Thermo Scientific K-Alpha+, Thermo Fisher Scientific, East Grinstead, UK) with Al *K*α X-ray (hν = 1486.6 eV). Prior to the high-resolution scan, the sample surface was sputter-cleaned with an Ar^+^ ion beam for 3 min to remove surface contaminants and the C 1*s* peak at 284.8 eV was used for calibration. A steady-state fluorescence spectrometer (Edinburgh FLS1000, Edinburgh Instruments, Livingston, UK) was used to test the photoluminescence (PL) spectra of the samples at room temperature with a 263 nm excitation light. The absorption coefficient of the β-Ga_2_O_3_ films and its relationship with the incident wavelength were measured using a spectroscopic ellipsometry (SE, RC2D, J.A. Woollam, Lincoln, NE, USA) to obtain information on the optical bandgap (*E*g) of the samples. The photoelectric characteristics of the fabricated photodetectors, including photocurrent, dark current, temporal photoresponse, and spectral responsivity, were measured using a self-assembled optoelectronic testing platform with a Keithley 4200 digital source meter (Tektronix, Cleveland, OH, USA), probe station, 150 W Xenon lamp, oscilloscope (Siglent SDS2102, Siglent Technologies, Shenzhen, China), black box, and monochromator. To calibrate and measure the photocurrent and responsivity of the fabricated photodetectors, a standard silicon photodetector was used for calibration.

## 3. Results and Discussion

### 3.1. Materials Characterization

XRD was performed to characterize the phase composition of the Ga_2_O_3_ thin films grown on different substrates by PECVD, and the results are shown in Figure 1c. All three samples exhibit a characteristic diffraction peak of the Si (400) crystal plane at 69.2° (JCPDS No. 27-1402), while the peak at 61.7° corresponds to a Cu *K*_β_ satellite peak. According to the standard card of β-Ga_2_O_3_ (JCPDS No. 00-043-1012), all three samples display clear characteristic diffraction peaks of β-Ga_2_O_3_, and the diffraction characteristics exhibit a significant substrate dependence. The diffraction patterns of samples S-n-Si and S-p-Si are highly consistent, showing three diffraction peaks at 18.9°, 38.3°, and 59.1°, which correspond to the (−201), (−402), and (−603) crystal planes of β-Ga_2_O_3_, respectively. This indicates that both samples possess a preferred orientation along the (−201) direction. There are no notable differences in peak intensity or full width at half maximum (FWHM) between these two samples, suggesting that the substrate doping type (n-type or p-type) does not significantly influence the crystal growth behavior of Ga_2_O_3_ thin films under the present process conditions. In contrast, the diffraction characteristics of sample S-p-Si/SiO_2_ are markedly different from those of the samples on bare Si substrates. In addition to the (−201), (−402), and (−603) series of diffraction peaks, multiple additional characteristic peaks of β-Ga_2_O_3_ appear at 30.3°, 33.4°, 35.2°, 42.9°, 45.8°, 48.6°, and 60.9°, corresponding to the (−401), (−111), (111), (311), (−312), (510), and (−711) crystal plane of β-Ga_2_O_3_, respectively. This indicates that sample S-p-Si/SiO_2_ features a typical random-orientation polycrystalline structure. This can be attributed to the fact that the amorphous SiO_2_ buffer layer completely shields the lattice-orientation guidance effect of the single-crystalline Si substrate during the initial deposition stage, rendering the nucleation barriers for various crystal orientations nearly equal. Consequently, the grains can develop freely during subsequent growth, ultimately resulting in a randomly oriented polycrystalline structure. Meanwhile, sample S-p-Si/SiO_2_ exhibits significantly enhanced peak intensities and notably narrowed FWHM values for almost all diffraction peaks. For example, for the representative (−402) diffraction peak, the FWHM decreases from 1.45° ± 0.02° for S-p-Si to 0.39° ± 0.02° for S-p-Si/SiO_2_. Using the Debye–Scherrer equation [19], *D* = *K*λ/(βcosθ), with *K* = 0.9 and λ = 1.5405 Å, the average crystallite sizes *D* were calculated to be 5.80 ± 0.50 nm for S-p-Si and 21.70 ± 0.50 nm for S-p-Si/SiO_2_, indicating that the introduction of the SiO_2_ buffer layer improves the crystalline quality of the Ga_2_O_3_ thin films and promotes grain growth.

**Figure 2:**
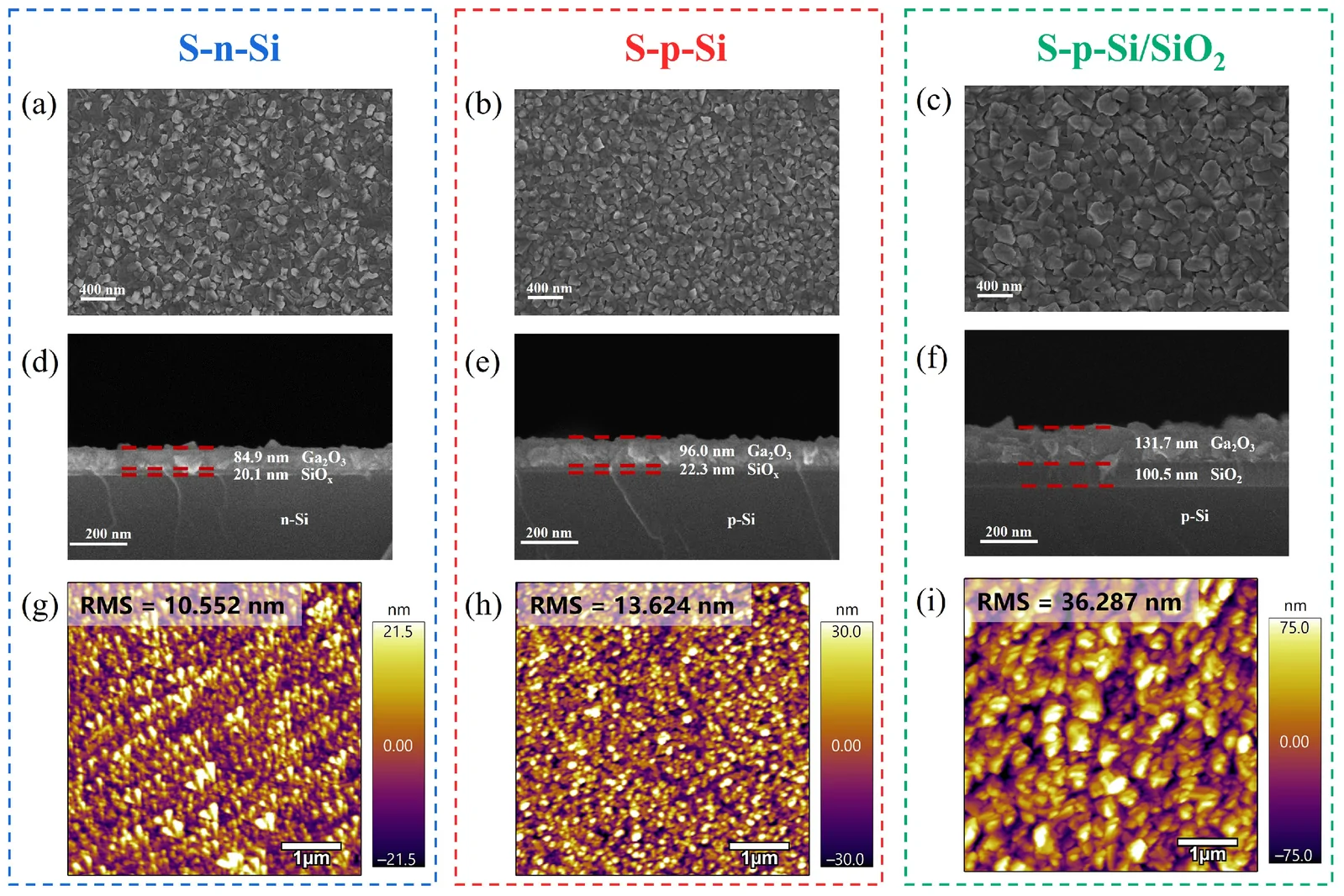
(**a**–**c**) Top-view SEM images, (**d**–**f**) cross-sectional SEM images, and (**g**–**i**) AFM images of Ga_2_O_3_ thin films prepared on different substrates by PECVD. The blue, red, and green dashed frames denote the samples S-n-Si, S-p-Si, and S-p-Si/SiO_2_, respectively.

SEM was employed to observe the surface morphology and cross-sectional structure of the synthesized Ga_2_O_3_ thin films, and the results are shown in Figure 2a–f. As shown in Figure 2a–c, all three samples exhibit continuous and dense film formation without macroscopic defects such as through-hole voids or cracks, indicating that the PECVD process possesses good film-forming compatibility on different substrates. The surface morphologies of samples S-n-Si and S-p-Si are highly similar, both consisting of nanocrystalline grains with a wide size distribution (20–150 nm), exhibiting a characteristic mixture of large and small grains with moderate surface flatness. In contrast, sample S-p-Si/SiO_2_ shows fully grown grains ranging from 100 to 300 nm, which are polygonal and equiaxed in shape, with clear and sharp grain boundaries and significantly improved size uniformity, leading to a more compact film structure. This difference primarily stems from the release of interfacial stress [16]. The amorphous SiO_2_ buffer layer acts as a compliant interlayer that possibly relieves the mismatch-induced stress and reduces the substrate-imposed constraint on grain growth, thereby providing favorable conditions for the full development of grains.

Cross-sectional SEM images (Figure 2d–f) show that the Ga_2_O_3_ thin films of all three samples grow uniformly along the substrate normal direction, forming continuous and dense layers. The film thicknesses of samples S-n-Si and S-p-Si are 84.9 nm and 96.0 nm, respectively, exhibiting little difference. Notably, between the Ga_2_O_3_ film and the substrate in both samples, a uniform thin layer with a thickness of approximately 20 nm is present. Based on its SEM contrast and previous reports [5,6,7,8,20], this thin layer is tentatively assigned to a SiO*_x_*-like (*x* ≤ 2) interfacial layer formed during the initial deposition stage. This is because at the early stage of PECVD growth, the highly reactive oxygen plasma species in the chamber rapidly react with the Si substrate surface, forming a loose SiO*_x_* layer. Thereafter, Ga and O active species begin to steadily deposit on the SiO*_x_* layer surface to form the Ga_2_O_3_ film. In contrast, sample S-p-Si/SiO_2_ exhibits a clear three-layer structure (p-Si substrate/SiO_2_ buffer layer/Ga_2_O_3_ film) with no additional secondary oxide layer. The Ga_2_O_3_ film thickness is approximately 131.7 nm, which is significantly larger than that of the samples on bare Si substrates. This directly verifies the crucial interfacial regulation role of the thermally oxidized SiO_2_ layer: the dense thermally oxidized SiO_2_ layer completely blocks the contact between oxygen plasma and the Si substrate, fundamentally suppressing the interfacial secondary oxidation reaction during the initial deposition stage. Consequently, the uncontrollable insulating interface layer present on bare Si substrates is eliminated, achieving precise control over the interfacial structure of the Ga_2_O_3_ thin film.

AFM was employed to characterize the surface morphology and root mean square (RMS) roughness of the three samples, and the results are shown in Figure 2g–i. All three samples exhibit continuous and intact surfaces without macroscopic defects. The surface morphologies of samples S-n-Si and S-p-Si are highly similar, with RMS values of 10.552 nm and 13.624 nm, respectively, showing no significant difference. In contrast, sample S-p-Si/SiO_2_ displays significantly larger surface grain protrusions, with an RMS value of 36.287 nm, which is much higher than those of the samples grown on bare Si substrates. The significant increase in RMS for sample S-p-Si/SiO_2_ arises from the full growth of grains and the transition of growth mode. First, as confirmed by the XRD and SEM results above, the introduction of the SiO_2_ buffer layer possibly relieves the mismatch-induced stress in Ga_2_O_3_ films on Si, promoting an increase in Ga_2_O_3_ grain size. The vertical height of the grains increases synchronously with their lateral dimensions, directly enlarging the surface height variation. Second, the amorphous SiO_2_ buffer layer effectively shields the orientation-guiding effect of the single-crystalline Si substrate on the film. The growth mode shifts from the high-density nucleation and synchronous growth mode on the bare Si substrate to a low-density nucleation and grain coalescence mode. The grain boundary steps and size hierarchies formed during grain coalescence further intensify surface undulations, ultimately resulting in RMS being significantly increased. It should be noted that this increase in RMS caused by grain growth does not indicate a degradation of film quality. On the contrary, it is accompanied by a reduction in grain boundary density and a decrease in bulk defects, which is beneficial for suppressing the dark current of the subsequent photodetector and provides a favorable structural basis for device performance optimization.

**Figure 3:**
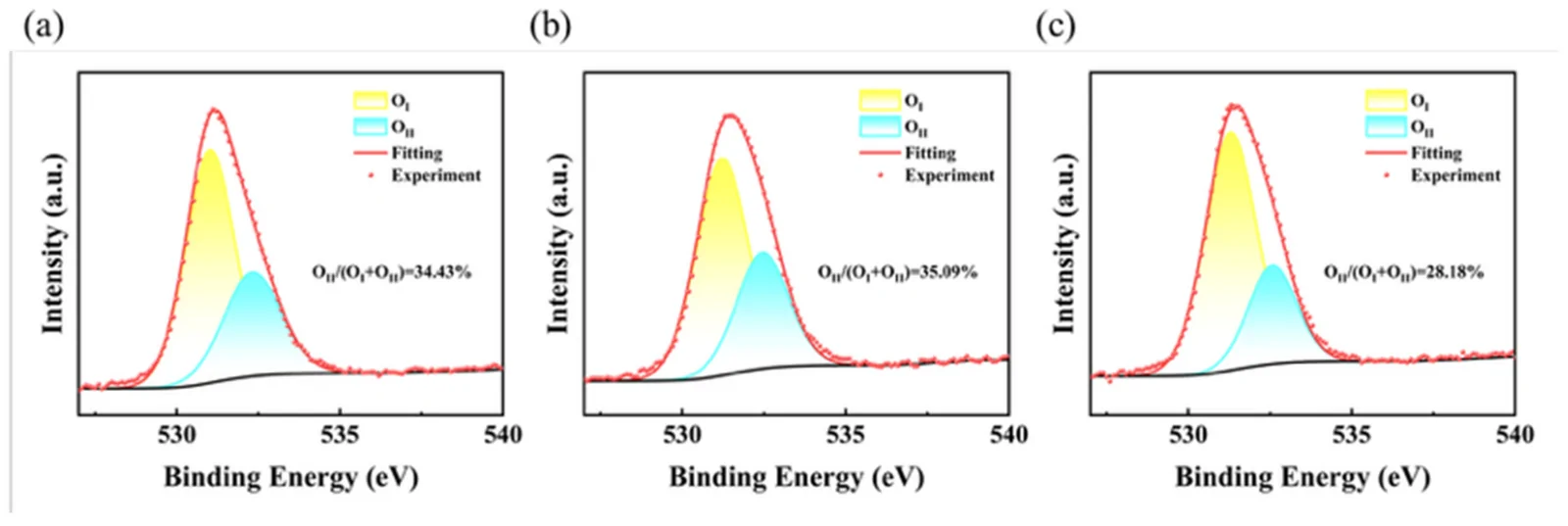
Deconvoluted O 1*s* XPS spectra of (**a**) S-n-Si, (**b**) S-p-Si, and (**c**) S-p-Si/SiO_2_.

XPS measurements were performed to analyze the chemical states of oxygen and evaluate the relative concentration of oxygen-vacancy-related defects in Ga_2_O_3_ films. Figure 3a–c shows the O 1*s* spectra of the Ga_2_O_3_ films on different substrates. Each O 1*s* spectrum can be deconvoluted into two components: O_I_ at 531.02 eV, assigned to Ga–O chemical bonds (lattice oxygen) in the film, and O_II_ at 532.32 eV, assigned to O^2-^ ions associated with oxygen vacancies (or defect-related oxygen species) in the film [19,21]. The area of each peak reflects the relative concentration of the corresponding component, and the O_II_/(O_I_ + O_II_) ratio represents the relative concentration of oxygen-vacancy defects. As shown in the figure, the calculated O_II_/(O_I_ + O_II_) ratio decreases from 35.09% for S-p-Si to 28.18% for S-p-Si/SiO_2_, suggesting a pronounced reduction in oxygen-vacancy concentration when the SiO_2_ buffer layer is introduced. This result indicates that introducing the SiO_2_ buffer layer significantly reduces the oxygen-vacancy defect concentration in the Ga_2_O_3_ film, which is expected to be beneficial for suppressing defect-related leakage pathways and improving the interfacial quality of the device.

**Figure 4:**
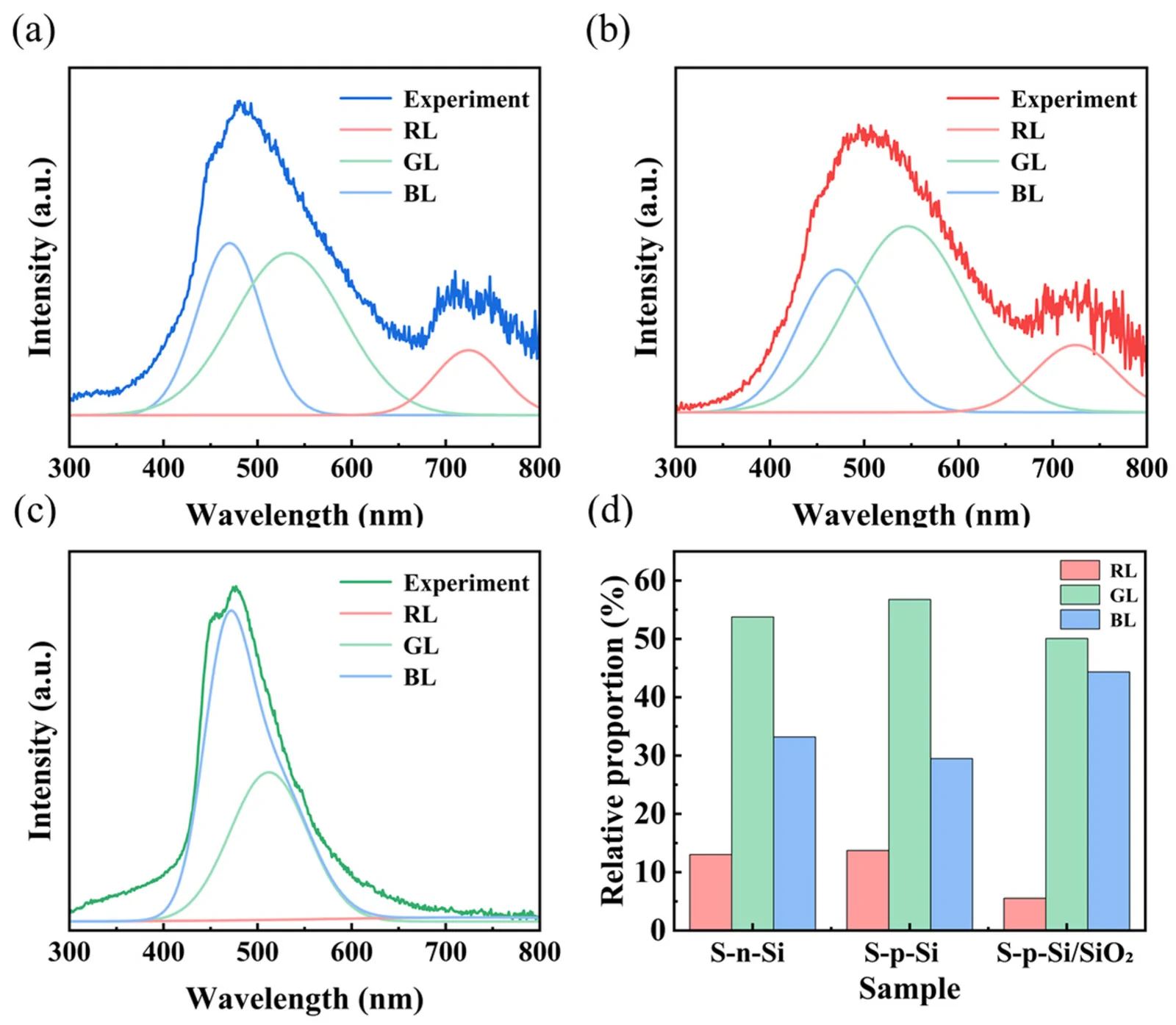
Gaussian deconvolution fitting results of the PL spectra for Ga_2_O_3_ films on different substrates: (**a**) S-n-Si, (**b**) S-p-Si, and (**c**) S-p-Si/SiO_2_. (**d**) Relative integrated area proportions of the blue (BL), green (GL), and red (RL) emission bands in the three samples.

To deeply reveal the defect luminescence mechanisms and their potential impact on photodetection performance, the room-temperature PL spectra of the Ga_2_O_3_ films on different substrates were measured and deconvoluted using multi-peak Gaussian fitting. As shown in Figure 4a–c, the broad emission spectra of all samples can be resolved into three characteristic bands: a blue (BL, 456–467 nm), green (GL, 516–538 nm), and red (RL, >600 nm) region. According to the defect luminescence theory of β-Ga_2_O_3_, the BL band is commonly attributed to donor–acceptor pair recombination involving oxygen vacancies as donors and gallium vacancies or Ga–O vacancy pairs as acceptors; the GL band is generally associated with deep-level defects involving oxygen vacancies and/or gallium vacancies; and the RL band is ascribed to recombination involving more complex deep-level defects, such as oxygen-vacancy clusters [22,23].

Figure 4d further quantitatively analyzes the relative integrated area proportions of the three emission bands. It is clearly observed that the substrate type significantly modulates the relative distribution of defect emissions. For the samples grown directly on n-Si and p-Si, the GL emission is dominant (accounting for ~53.76% and ~56.76%, respectively), accompanied by a notable proportion of RL emission (~13.04% and 13.74%). After introducing the SiO_2_ buffer layer (S-p-Si/SiO_2_), the complex deep-level defects are effectively suppressed, as evidenced by the dramatic reduction in the RL proportion (dropping to 5.53%). Meanwhile, the proportion of the BL component significantly increases (from 29.49% to 44.34%).

This phenomenon indicates that the SiO_2_ buffer layer not only passivates the interface but more importantly, effectively suppresses the formation of complex deep-level defect clusters (corresponding to the significant reduction in RL), thereby improving the microstructural quality of the Ga_2_O_3_ film. A higher density of such complex defects in the n-Si and p-Si based samples would act as non-radiative recombination centers and leakage paths, which is detrimental to the photodetector’s signal-to-noise ratio. Consequently, the introduction of the SiO_2_ buffer layer is beneficial for enhancing the photoresponse performance of the device by passivating interface states and reducing defect-induced recombination losses.

**Figure 5:**
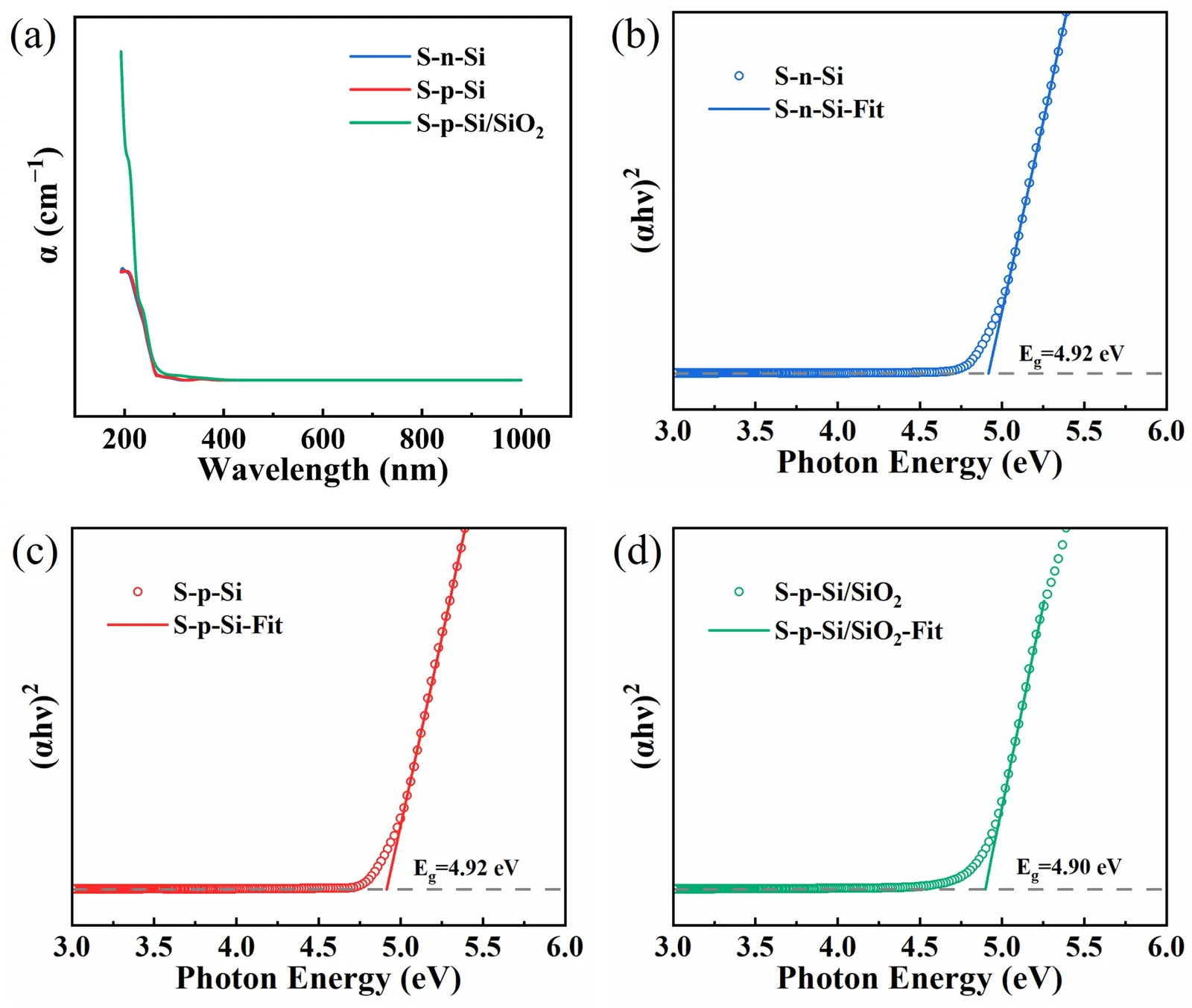
(**a**) Absorption coefficient versus wavelength; (**b**–**d**) Tauc plots of Ga_2_O_3_ thin films prepared on different types of substrates by PECVD.

The optical absorption properties of the three Ga_2_O_3_ thin films were characterized by SE. The variation of the absorption coefficient (α) with wavelength is shown in Figure 5a. All three samples exhibit a sharp intrinsic absorption edge near 250 nm. The absorption coefficient rapidly decays to near zero for wavelengths longer than 300 nm, and no notable sub-bandgap absorption is observed in the visible to near-infrared region, indicating the absence of significant deep-level defects. The absorption coefficient curves of samples S-n-Si and S-p-Si almost completely overlap, suggesting that the substrate doping type has no significant effect on the intrinsic optical absorption of the synthesized Ga_2_O_3_ films. In contrast, the absorption coefficient of sample S-p-Si/SiO_2_ is significantly higher than that of the samples grown on bare Si substrates, and the absorption edge is steeper. This is attributed to its higher crystalline quality and fewer grain boundary defects, leading to more efficient intrinsic interband absorption. The *E*g of each sample was determined by applying the Tauc plot method to extrapolate the linear region of the curves (Figure 5b–d) [24]. The accuracy of the absorption coefficient α is critical for reliable Tauc analysis, because sub-bandgap absorption and the choice of the linear fitting region can significantly affect the extracted optical bandgap [25,26,27]. In this work, α was extracted from SE using a Tauc–Lorentz oscillator model, which provides a self-consistent determination of the refractive index and extinction coefficient. To minimize the contribution of defect-related sub-bandgap absorption, the linear fit was restricted to the steeply rising region corresponding to absorption coefficients above 10^4^ cm^−1^, where band-to-band transitions dominate. The fitting ranges and coefficients of determination (*R*^2^ > 0.99) are summarized in Table 1. Furthermore, Urbach tail analysis was performed to distinguish fundamental band-edge absorption from defect-related sub-bandgap absorption, yielding Urbach energies of 151 meV for S-n-Si, 108 meV for S-p-Si, and 288 meV for S-p-Si/SiO_2_. Based on this rigorous fitting procedure, the extracted bandgap values were 4.92 eV for both S-n-Si and S-p-Si, and 4.90 eV for S-p-Si/SiO_2_. It should be noted that the Tauc method has inherent limitations, including sensitivity to fitting range selection and the presence of absorption tails [25,26,27]. Nevertheless, the consistent bandgap values (4.90–4.92 eV) obtained here agree well with the accepted intrinsic bandgap of *β*-Ga_2_O_3_ (4.90 eV), supporting the reliability of our analysis.

### 3.2. Optoelectronic Performance Analysis

To evaluate the electrical performance of Ga_2_O_3_ thin films grown on different silicon-based substrates for solar-blind ultraviolet photodetection applications, MSM photodetectors with an interdigitated electrode structure were fabricated based on the three sets of samples. Systematic optoelectronic tests were conducted on the I–V characteristics, spectral responsivity, transient response, and multi-cycle stability of the devices.

**Figure 6:**
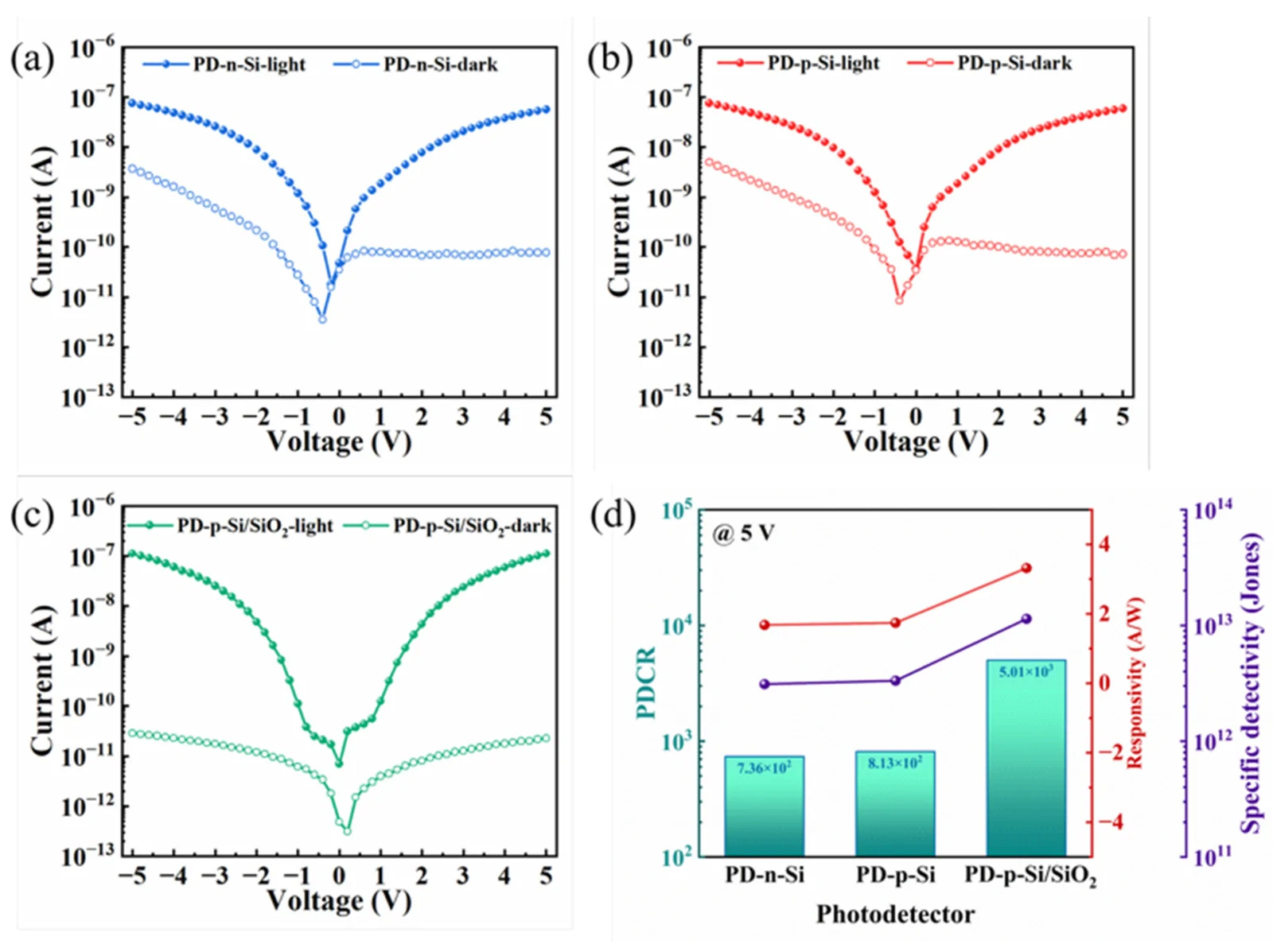
I–V characteristic curves of (**a**) PD-n-Si, (**b**) PD-p-Si, and (**c**) PD-p-Si/SiO_2_ under dark and illumination conditions (254 nm, 400 μW/cm^2^). (**d**) Summary of the photo-to-dark current ratio, responsivity, and theoretical shot-noise-limited specific detectivity for the three fabricated devices at a bias of 5 V.

Figure 6a–c present the I–V characteristic curves of the three fabricated devices under dark and illuminated conditions, respectively. The photocurrent was measured under 254 nm ultraviolet illumination with an intensity of 400 μW/cm^2^, while the dark current was recorded in the absence of light, with the bias voltage ranging from −5 V to 5 V. It can be observed that under 254 nm UV illumination, the current of all three devices increases significantly, with the photocurrent being much higher than the dark current. This indicates that all three devices possess good ultraviolet photoresponse characteristics and can effectively achieve solar-blind ultraviolet detection. As shown in Figure 6a and Figure 4b, the I–V characteristic curves of PD-n-Si and PD-p-Si are highly similar. The dark I–V curves exhibit pronounced asymmetry: under negative bias, the dark current increases with the absolute value of the bias voltage, while under positive bias, the dark current saturates at around 0.5 V. The asymmetric I–V behavior observed for devices on bare Si substrates may originate from a combination of the following mechanisms, including inhomogeneous Schottky barrier heights at the Ni/Ga_2_O_3_ interface, contact inhomogeneity and interface states, substrate-assisted leakage through the thin interfacial layer on bare Si, and inhomogeneous distribution of oxygen vacancy defects within the films [28,29]. In contrast, as shown in Figure 6c, the I–V characteristic curves of PD-p-Si/SiO_2_ are markedly different from those of the devices on bare Si substrates. Specifically, the dark current of this device is significantly reduced, and the dark I–V curve exhibits excellent symmetry.

The photo-to-dark current ratio (PDCR) [30], responsivity (*R*) [19], and theoretical shot-noise-limited specific detectivity (D*) [31] can be calculated using Equations (1)–(3), respectively.

(1) $$\mathrm{PDCR}=\frac{I_{\mathrm{ph}}}{I_{d}}=\frac{I_{p}-I_{d}}{I_{d}}$$

(2) $$R=\frac{I_{\mathrm{ph}}}{{\mathrm{SP}}_{\lambda }}$$

(3) $$D^{*}=R\sqrt{\frac{S}{2qI_{d}}}$$

where *I_ph_* is the photocurrent; *I_p_* is the current under illumination; *I_d_* is the current in the dark; *S* is the effective illuminated area (in our work, *S* = 8500 μm^2^); *P_λ_* is the light intensity of the specific wavelength; and *q* is the elementary charge. It should be noted that Equation (3) assumes shot-noise-limited operation and ignores 1/f and thermal noise; the calculated D* is therefore a theoretical shot-noise-limited value rather than an experimentally measured detectivity. Figure 6d summarizes the calculated photo-to-dark current ratio, responsivity, and theoretical shot-noise-limited specific detectivity for the three fabricated devices at a bias of 5 V. It can be observed that for PD-n-Si, PD-p-Si, and PD-p-Si/SiO_2_, the photo-to-dark current ratios are 7.36 × 10^2^, 8.13 × 10^2^, and 5.01 × 10^3^, respectively; the responsivities are 1.68, 1.74, and 3.32 A/W, respectively; and the specific detectivities are 3.11 × 10^12^, 3.33 × 10^12^, and 1.14 × 10^13^ Jones, respectively. Compared with the other two devices, PD-p-Si/SiO_2_ exhibits enhanced PDCR, *R*, and D*, ranking the highest among the three fabricated devices. This indicates that this device possesses superior dark current suppression capability, higher photoelectric conversion efficiency, and stronger weak-light detection sensitivity, demonstrating significant performance advantages in solar-blind ultraviolet detection applications.

**Figure 7:**
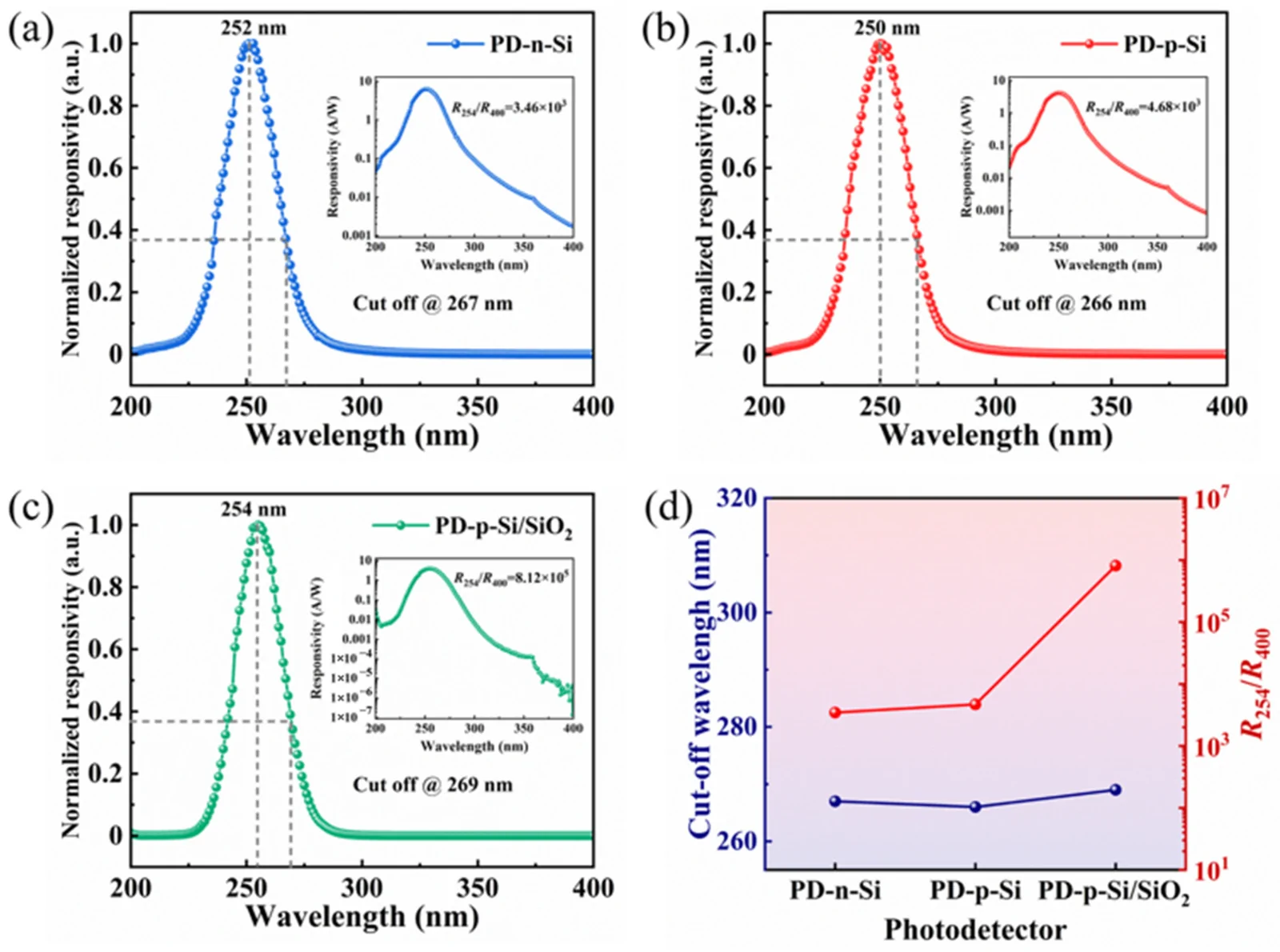
Normalized spectral responsivity curves of (**a**) PD-n-Si, (**b**) PD-p-Si, and (**c**) PD-p-Si/SiO_2_ under a 5 V bias and 400 μW/cm^2^ illumination, where the dotted lines indicate the cutoff wavelengths (insets show the UV-to-visible rejection ratio for the three fabricated devices). (**d**) Summary of the cut-off wavelength and UV-to-visible rejection ratio for the three fabricated devices.

Figure 7a–c presents the normalized spectral responsivity curves of the three fabricated devices under a 5 V bias and a light intensity of 400 μW/cm^2^. All three devices exhibit significant photoresponse near 254 nm, and the cut-off wavelengths (where responsivity declines to 1/e of its peak [32]) are 267, 266, and 269 nm, respectively. This indicates that the response bands of the three devices fully cover the solar-blind region, endowing them with excellent solar-blind ultraviolet detection capability and effective avoidance of background noise interference in the visible band. To further quantitatively analyze the spectral selectivity of the devices, the responsivity ratio at 254 nm and 400 nm (*R*_254_/*R*_400_) is used as the UV-to-visible rejection ratio. As shown in the insets of Figure 7a–c, the UV-to-visible rejection ratios of PD-n-Si, PD-p-Si, and PD-p-Si/SiO_2_ are 3.46 × 10^3^, 4.68 × 10^3^, and 8.12 × 10^5^, respectively. It can be observed that PD-p-Si/SiO_2_ exhibits a significantly enhanced UV-to-visible rejection ratio, indicating the best spectral selectivity. This is because the introduction of the SiO_2_ buffer layer substantially improves the crystalline quality of the Ga_2_O_3_ thin film, reducing the density of defect states and grain boundary defects within the film. The photoresponse in the visible band mainly originates from defect-assisted sub-bandgap absorption. The improved crystalline quality effectively suppresses sub-bandgap absorption, thereby reducing the responsivity at 400 nm. In contrast, the photoresponse in the deep ultraviolet band at 254 nm arises from the intrinsic interband transition of Ga_2_O_3_, which is less affected by defect states. Consequently, the UV-to-visible rejection ratio is significantly improved, and the spectral selectivity of the device is optimized for PD-p-Si/SiO_2_.

**Figure 8:**
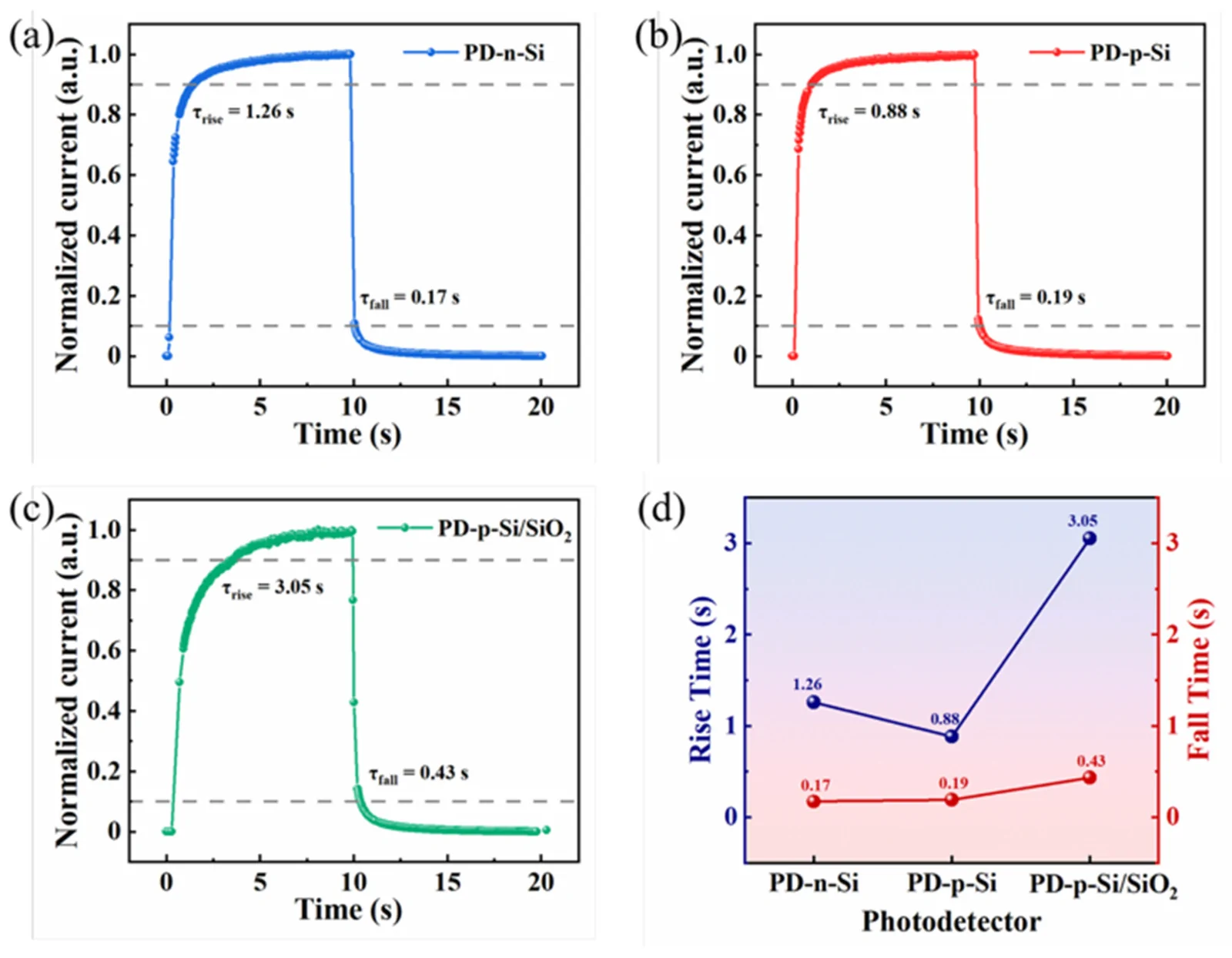
Normalized transient response curves of (**a**) PD-n-Si, (**b**) PD-p-Si, and (**c**) PD-p-Si/SiO_2_ under a 5 V bias and 254 nm illumination (400 μW/cm^2^). (**d**) Summary of the rise and fall times for the three fabricated devices.

To characterize the response speed of the devices to incident light signals, transient response measurements were performed on the three fabricated devices under a 5 V bias by turning the incident light on and off. The test results are shown in Figure 8a–c. The current was normalized to the [0, 1] range. The rise time (τ_rise_) was defined as the time interval from 10% to 90% of the rising edge of the transient response curve, and the fall time (τ_fall_) was defined as the time interval from 90% to 10% of the decaying edge. The obtained rise/fall times for PD-n-Si, PD-p-Si, and PD-p-Si/SiO_2_ are 1.26/0.17 s, 0.88/0.19 s, and 3.05/0.43 s, respectively. A summary of the response times for the three devices is shown in Figure 8d. It can be observed that the response times of PD-n-Si and PD-p-Si are similar, with PD-p-Si exhibiting a slightly faster rise time than PD-n-Si. In contrast, both the rise time and fall time of PD-p-Si/SiO_2_ are significantly longer than those of the other two devices, indicating the slowest response speed.

**Figure 9:**
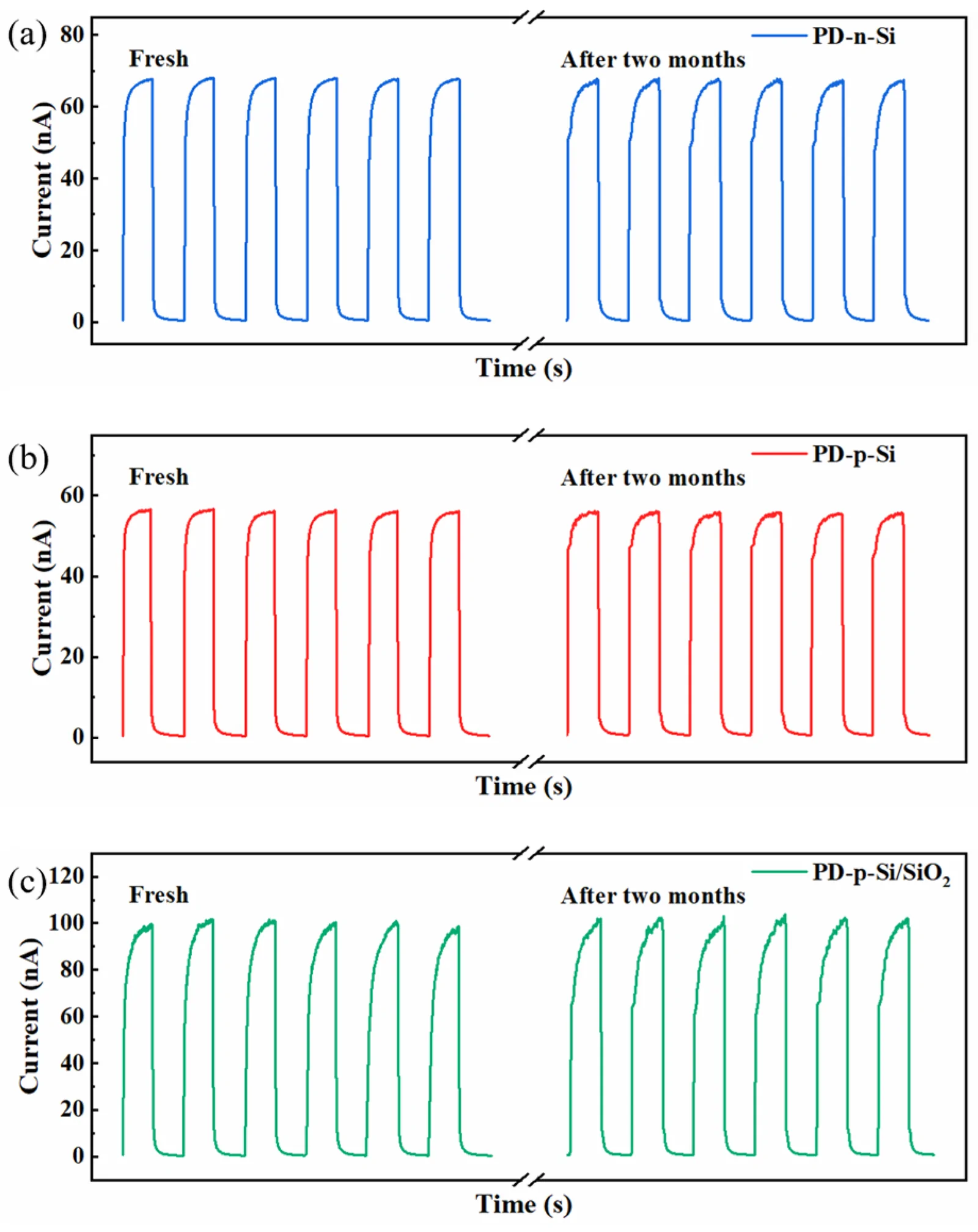
Multi-cycle I–t characteristic curves of (**a**) PD-n-Si, (**b**) PD-p-Si, and (**c**) PD-p-Si/SiO_2_ measured immediately after fabrication and after two months of storage in ambient atmosphere, under alternating 254 nm illumination (400 μW/cm^2^) at a 5 V bias.

To evaluate the stability and repeatability of the three devices, multi-cycle I–t characteristic tests were performed on the freshly fabricated devices and on the devices after storage in ambient atmosphere for two months. The results are presented in Figure 9. It can be observed that during continuous multi-cycle switching tests under 254 nm solar-blind ultraviolet illumination, the photocurrent and dark current levels of each device in each cycle are highly consistent, with no noticeable current degradation, waveform distortion, or performance fluctuation. This indicates that all three devices possess good multi-cycle operational stability and response repeatability, maintaining stable detection performance under continuous on-off switching conditions. Moreover, the I–t characteristic curves measured after two months of storage show negligible performance degradation, demonstrating that the devices exhibit good durability in ambient atmosphere and are not easily affected by the surrounding environment. This further verifies the favorable long-term reliability of the devices.

Based on the above optoelectronic test results, the key performance parameters of the fabricated three photodetectors are summarized in Table 2. It can be clearly observed that the performance of PD-n-Si and PD-p-Si is highly similar, while the performance of PD-p-Si/SiO_2_ exhibits significant differences. Specifically, for PD-p-Si/SiO_2_, the dark current is greatly reduced, the photo-to-dark current ratio and theoretical shot-noise-limited specific detectivity are significantly enhanced, and the spectral selectivity is improved, but the response time is prolonged. These experimental results indicate that the substrate doping type (n-type, p-type) has a weak effect on device performance, whereas the introduction of the SiO_2_ buffer layer is the key factor leading to the performance differences.

To place the present results in context, the key performance metrics of the fabricated devices are compared with recently reported Ga_2_O_3_/Si solar-blind photodetectors in Table 3, including devices employing AlN, Al_2_O_3_, and SiO_2_ buffer layers. While some high-gain devices (e.g., APDs) exhibit higher absolute responsivity under high reverse biases, the performance of our MSM device is highly competitive, particularly in terms of PDCR and detectivity at a low operating bias of 5 V. The main advance of this work lies in the combination of low-cost PECVD growth, a CMOS-compatible thermally oxidized SiO_2_/Si substrate, and balanced PDCR, responsivity, detectivity, and spectral selectivity, without additional buffer deposition equipment.

### 3.3. Mechanism Analysis

To gain a deeper understanding of the mechanism by which the introduction of a SiO_2_ buffer layer influences the growth of Ga_2_O_3_ thin films and the performance of corresponding MSM photodetectors, a systematic analysis is conducted in this subsection based on the material characterization and device performance results, combined with schematic diagrams of the energy band structure and carrier transport pathways. As revealed by the data analysis in the previous two subsections, the substrate doping type has a relatively weak effect on the growth of Ga_2_O_3_ thin films and the performance of the resulting photodetectors. This indicates that the underlying mechanisms of bare Si substrates with different doping types are similar. Therefore, the samples on p-Si substrates are taken as an example for discussion.

**Figure 10:**
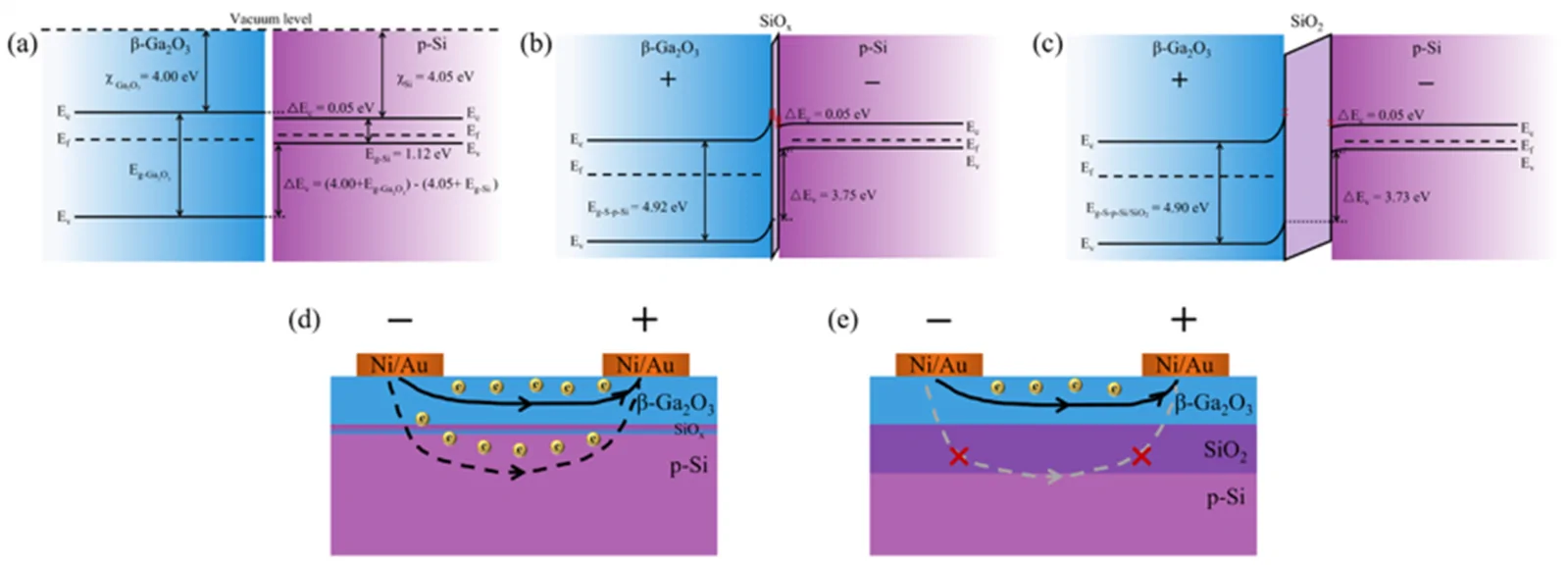
(**a**) Schematic energy band diagram of Ga_2_O_3_ before contact with p-Si. (**b**,**c**) Schematic energy band diagrams of PD-p-Si (**b**) and PD-p-Si/SiO_2_ (**c**) under dark conditions. (**d**,**e**) Schematic diagrams of carrier transport pathways in PD-p-Si (**d**) and PD-p-Si/SiO_2_ (**e**).

According to the Anderson energy band model, a schematic diagram of the energy band structure of Ga_2_O_3_ and Si before contact is depicted, as shown in Figure 10a. The conduction band offset (∆*E*_C_) and valence band offset (∆*E*_V_) between Ga_2_O_3_ and the Si substrate can be calculated using Equation (4) and Equation (5), respectively [7,35]:

(4) $$∆E_{C}=\chi_{\mathrm{Si}}-\chi_{{\mathrm{Ga}}_{2}O_{3}}$$

(5) $$∆E_{V}=\left(\chi_{{\mathrm{Ga}}_{2}O_{3}}+E_{g-{\mathrm{Ga}}_{2}O_{3}}\right)-(\chi_{\mathrm{Si}}+E_{g-\mathrm{Si}})$$

where *χ_Si_* is the electron affinity of Si, taken as 4.05 eV; $\chi_{{\mathrm{Ga}}_{2}O_{3}}$ is the electron affinity of Ga_2_O_3_, taken as 4.00 eV; $E_{g-{\mathrm{Ga}}_{2}O_{3}}$ is the bandgap of Ga_2_O_3_, which is 4.92 eV for sample S-p-Si and 4.90 eV for sample S-p-Si/SiO_2_; and *E_g_*_−*Si*_ is the bandgap of Si, taken as 1.12 eV. The calculated conduction band offsets ∆*E*_C_ are both 0.05 eV, while the valence band offsets ∆*E*_V_ are 3.75 eV and 3.73 eV, respectively.

When no voltage is applied, the device as a whole is in a thermal equilibrium state, with the Fermi levels of Ga_2_O_3_ and p-Si aligned, leading to band bending in the conduction and valence bands. When a voltage is applied, the bias is applied between the two Ni/Au electrodes on top of β-Ga_2_O_3_, while the p-Si substrate is electrically floating and at a relatively low potential. The side of Ga_2_O_3_ under positive bias has a relatively higher potential, which further intensifies the band bending, resulting in the energy band structure illustrated in Figure 10b,c. For device PD-p-Si, as shown in Figure 10b, a thin and defective interfacial layer tentatively assigned as SiO*_x_*-like (*x* ≤ 2) exists between Ga_2_O_3_ and p-Si. This allows electrons from the Ga_2_O_3_ side to tunnel through these defect states into the Si substrate, forming a vertical leakage current. Consequently, under dark conditions, PD-p-Si exhibits significant interfacial leakage, which accounts for its relatively high dark current. For device PD-p-Si/SiO_2_, as shown in Figure 10c, the introduced 100 nm thermally oxidized SiO_2_ layer is dense and contains few intrinsic defects. This layer forms a very high energy barrier between Ga_2_O_3_ and p-Si, making it impossible for either electrons or holes to overcome this barrier via thermionic emission or tunneling. Moreover, the sufficiently thick SiO_2_ layer physically blocks the vertical carrier transport path. Therefore, under dark conditions, the leakage current originating from the Si substrate in PD-p-Si/SiO_2_ is completely suppressed, leading to a significantly reduced dark current.

Based on the above analysis and previous literature, two carrier transport pathways exist in the devices, as illustrated in Figure 10d,e [6,12,33]. The first pathway is lateral transport within the β-Ga_2_O_3_ layer, and the second is vertical transport from the β-Ga_2_O_3_ layer through the SiO*_x_* layer into the Si substrate, followed by lateral transport within the substrate. As shown in Figure 10d, the SiO*_x_* layer is an uncontrollable interfacial secondary oxide layer formed during the initial film deposition stage. It is structurally loose and contains numerous intrinsic defects such as dangling bonds, oxygen vacancies, and micropores, resulting in a large interfacial leakage current between Ga_2_O_3_ and the Si substrate. Moreover, it cannot fully buffer the lattice mismatch stress between Ga_2_O_3_ and the Si substrate, which limits grain growth and introduces a large number of lattice defects into the film, further increasing defect-assisted tunneling current. Together with the interfacial leakage current, these factors lead to an increase in dark current for PD-p-Si [36].

In contrast, as shown in Figure 10e, the device with the SiO_2_ buffer layer exhibits a different carrier transport pathway. The SiO_2_ buffer layer is prepared by in situ thermal oxidation, resulting in a dense structure with low intrinsic defect density. Its thickness of 100 nm is sufficient to effectively block vertical carrier transport, fundamentally eliminating the interfacial leakage current between Ga_2_O_3_ and the Si substrate. Carrier transport mainly relies on the first (lateral) pathway, significantly reducing the dark current and achieving a substantial increase in the photo-to-dark current ratio. At the same time, the dense SiO_2_ layer also acts as a diffusion barrier, suppressing interdiffusion of Ga and Si elements and maintaining interfacial chemical stability. Its adequate thickness possibly relieves the mismatch-induced stress in Ga_2_O_3_ films on Si, promotes full grain growth, reduces lattice defects within the film, and diminishes the recombination loss of photogenerated carriers at grain boundaries and bulk defects, thereby improving carrier collection efficiency [37]. Consequently, the responsivity and theoretical shot-noise-limited specific detectivity are simultaneously enhanced for PD-p-Si/SiO_2_. Therefore, the introduction of the SiO_2_ buffer layer improves the overall device performance by cutting off the vertical leakage path of carriers to the Si substrate, releasing mismatch-induced stress in Ga_2_O_3_ films on Si, and suppressing interfacial defects. It should be noted that the two-path carrier-transport model proposed here is based on the dark-current behavior, the cross-sectional SEM observations, and previous literature, rather than on direct substrate-bias or vertical-transport measurements. Dedicated substrate-contact devices and substrate-bias experiments are required to quantitatively separate the lateral and vertical transport pathways, and this will be pursued in future work.

However, it is worthwhile mentioning that the response speed of PD-p-Si/SiO_2_ is slightly slower than that of PD-p-Si and PD-n-Si. The slower response of PD-p-Si/SiO_2_ is likely governed by a combination of mechanisms: (i) suppression of vertical transport through the high-mobility Si substrate, which eliminates a fast carrier extraction channel; (ii) trapping/detrapping processes associated with residual defects and grain boundaries in the Ga_2_O_3_ film; and (iii) possible contributions from interface states and persistent-photoconductivity-like effects [37,38,39,40]. Temperature-dependent transient and frequency-dependent measurements are required to quantitatively separate these contributions.

Finally, it is noteworthy that although the Ga_2_O_3_ film on SiO_2_/Si (131.7 nm) is thicker than those on bare Si (84.9 and 96.0 nm), the n-Si and p-Si devices with an ~11 nm thickness difference exhibit nearly identical performance. In contrast, the SiO_2_-buffered device shows a much lower dark current, a 6.2-fold higher PDCR, a 3.4-fold higher theoretical shot-noise-limited specific detectivity, and a 174-fold higher UV-to-visible rejection ratio (compared to the PD-p-Si device). A ~37% increase in thickness alone cannot account for these improvements; in particular, a thicker film would not be expected to suppress dark current. It should also be noted that the increased thickness may partially contribute to the higher responsivity through enhanced light absorption. Therefore, the dominant effect is attributed to the SiO_2_ buffer-induced improvement in crystalline quality and interfacial blocking.

## 4. Conclusions

In this work, β-Ga_2_O_3_ thin films were deposited by PECVD on n-Si(100), p-Si(100), and p-Si(100) substrates with a 100 nm thermally oxidized SiO_2_ layer on one side, and subsequently used to fabricate MSM solar-blind ultraviolet photodetectors with an interdigitated electrode structure. The results demonstrate that the introduction of the SiO_2_ buffer layer markedly enhances the crystalline quality and interfacial characteristics of the Ga_2_O_3_ thin films through mechanisms such as relieving the mismatch-induced stress in Ga_2_O_3_ films on Si, suppressing the formation of an additional interfacial layer tentatively assigned as SiO*_x_*-like (*x* ≤ 2), and reducing the initial nucleation density. Consequently, the corresponding device outperforms its counterparts on bare silicon substrates in key performance metrics including the photo-to-dark current ratio, responsivity, theoretical shot-noise-limited specific detectivity, and UV-to-visible rejection ratio, exhibiting superior solar-blind ultraviolet detection capability. However, the device response speed is slightly reduced, which is tentatively attributed to a combination of suppressed vertical carrier transport through the Si substrate, trapping/detrapping at defects and grain boundaries, and possible interface-state effects. A comprehensive comparison indicates that introducing a SiO_2_ buffer layer on silicon substrates is an effective strategy for optimizing the structural quality of Ga_2_O_3_ thin films and enhancing the overall optoelectronic performance of the devices, thereby providing a feasible approach and experimental basis for achieving silicon-integrated, high-performance Ga_2_O_3_ solar-blind ultraviolet photodetectors.

## Figures and Tables

**Table 1:** Summary of Tauc analysis parameters for the three samples.

Sample	Tauc Fitting Ranges (eV)	*R* ^2^	*Eg* (eV)
S-n-Si	5.02–5.39	0.99925	4.92
S-p-Si	5.10–5.39	0.99936	4.92
S-p-Si/SiO_2_	5.06–5.25	0.99721	4.90

**Table 2:** Summary of performance parameters for the three fabricated photodetectors.

Device	PDCR	*R* (A/W)	D* (Jones)	*R*_254_/*R*_400_	τ_rise_/τ_fall_ (s/s)
PD-n-Si	7.36 × 10^2^	1.68	3.11 × 10^12^	3.46 × 10^3^	1.26/0.17
PD-p-Si	8.13 × 10^2^	1.74	3.33 × 10^12^	4.68 × 10^3^	0.88/0.19
PD-p-Si/SiO_2_	5.01 × 10^3^	3.32	1.14 × 10^13^	8.12 × 10^5^	3.05/0.43

**Table 3:** Benchmarking of representative Ga_2_O_3_/Si solar-blind photodetectors.

Device Architecture	Growth Method	Buffer/Passivation Layer	Bias (V)	*R* (A/W)	PDCR	D* (Jones)	Response Time (s)	Ref.
β-Ga_2_O_3_/SiO_2_/p-Si MSM (This work)	PECVD	SiO_2_ (100 nm)	5	3.32	5.01 × 10^3^	1.14 × 10^13^	3.05/0.43	This work
β-Ga_2_O_3_/SiO_2_/n-Si nBn	MOCVD	SiO_2_ (50 nm)	−10	3.92 × 10^−2^	1.1 × 10^3^	4.8 × 10^12^	9.4 × 10^−3^ (decay)	[33]
β-Ga_2_O_3_/AlN/p-Si MSM	MOCVD	AlN	3	38.8	8.5 × 10^5^	2.27 × 10^15^	-	[12]
β-Ga_2_O_3_/AlN/n-Si APD	MOCVD	AlN (150 nm)	−55	131.3	-	2.9 × 10^15^	-	[32]
ϵ-Ga_2_O_3_/Al_2_O_3_/p-Si Heterojunction	MOCVD	Al_2_O_3_	−10	8.1	6.62 × 10^4^	4.21 × 10^13^	0.87/0.29	[15]
β-Ga_2_O_3_/p-Si Heterojunction	PLD	-	3	370	9.4 × 10^2^	-	1.79/0.27	[34]

## Data Availability

The original contributions presented in this study are included in the article. Further inquiries can be directed to the corresponding author.
